# Neurorestorative therapeutic strategies for sequela of central nervous system infections

**DOI:** 10.1016/j.imj.2024.100141

**Published:** 2024-10-11

**Authors:** Hongyun Huang, Paul R. Sanberg, Hari Shanker Sharma

**Affiliations:** a Beijing Rehabilitation Hospital, Capital Medical University, Beijing 100144, China; b Center of Excellence for Aging & Brain Repair, Department of Neurosurgery & Brain Repair, Morsani College of Medicine, University of South Florida, Tampa, FL 33612, USA; c Intensive Experimental CNS Injury and Repair, Department of Surgical Sciences, University Hospital, Uppsala University, Uppsala S-751 85, Sweden

There are a very significant number of papers reporting on the management of acute and sub-acute central nervous system (CNS) infections. As well as studies introducing manifestations or mechanisms of sequelae or complications following CNS infections, such as COVID-19 [[1], [2]]. However, there are relatively few reports on neurorestorative therapeutic strategies for addressing the sequelae of CNS infections. Many medical professionals still hold the belief that there are no effective therapies for restoring lost neurological functions in patients with sequelae of CNS infections.

The International Association of Neurorestoratology (IANR) states in their Beijing Declaration that functional recovery is possible after chronic CNS injury and neurodegeneration [3], including conditions caused by nervous system infections. Contrary to the traditional belief that CNS damage is irreparable, current research demonstrates that chronic stages or sequelae of CNS damage can be partially restored using neurorestorative therapeutic strategies [3], [4], [5]. This evolving perspective is transforming treatment approaches for patients with nervous system disease sequelae. Neurorestorative therapeutic methods include pharmaceutical interventions, electro-magnetic stimulation, brain-machine interface, cell therapies, immunotherapy, bioengineering, gene therapy, tissue engineering, restorative surgery, and combinations with classical rehabilitation practices [3].

Recent clinical studies have demonstrated the effectiveness of these neurorestorative strategies in improving neurological dysfunctions and enhancing the quality of life for patients with CNS damage sequelae [[6], [7], [8]].

We can show clinical neurorestorative treatment achievements using chronic complete spinal cord injury (SCI) as an example [9]. Nerve bridging could restore some neurological function in patients with complete chronic SCI. Epidural stimulation could activate nerve circuits and promote nerve remodeling and functional recovery in patients with complete SCI. Brain-computer interface and artificial neuroprostheses helped paralyzed patients carry out activities of daily living. Many kinds of cell transplantation including olfactory ensheathing cells, mesenchymal stromal cells, peripheral blood mononuclear cells, bone marrow and umbilical cord blood cells, bone marrow hematopoietic stem cells, Schwann cells, and embryonic stem cell products through intravascular infusion, intrathecal or intralesional injection, or multi-channel administration showed the improvements of both neurological functions (sensory and motor functions) and quality of life. Comprehensive application of several treatments including cell transplantation, nerve bridging, electrical stimulation, neurorehabilitation, and brain-computer interface gait training programs could achieve more neurological function recovery, such as standing and walking ability in patients with chronic complete SCI.Even patients with sequelae of either bacteria or virus infection in spinal cord, they also have chance to restore their lost neurological functions and improve their quality of life through cell therapy, neuromodulation, and selective dorsal (posterior) rhizotomy [10].

Given these developments, we strongly recommend that physicians explore neurorestorative therapy options for patients with sequelae of CNS infections, providing them with opportunities to improve their quality of life.

## Funding

None.

## CRediT authorship contribution statement

**Hongyun Huang:** Writing – original draft. **Paul R. Sanberg:** Writing – review & editing. **Hari Shanker Sharma:** Writing – review & editing.
